# Fluorescence-Guided High-Grade Glioma Surgery More Than Four Hours After 5-Aminolevulinic Acid Administration

**DOI:** 10.3389/fneur.2021.644804

**Published:** 2021-03-09

**Authors:** Georgios A. Maragkos, Alexander J. Schüpper, Nikita Lakomkin, Panagiotis Sideras, Gabrielle Price, Rebecca Baron, Travis Hamilton, Sameah Haider, Ian Y. Lee, Constantinos G. Hadjipanayis, Adam M. Robin

**Affiliations:** ^1^Department of Neurosurgery, Icahn School of Medicine at Mount Sinai, Mount Sinai Health System, New York, NY, United States; ^2^Department of Radiology, Icahn School of Medicine at Mount Sinai, Mount Sinai Health System, New York, NY, United States; ^3^Department of Neurosurgery, Henry Ford Health System, Detroit, MI, United States; ^4^Department of Neurosurgery, Icahn School of Medicine, Mount Sinai Beth Israel, Mount Sinai Health System, New York, NY, United States

**Keywords:** fluorescence, 5-ALA, glioma, glioblastomas, brain tumors, neuro-oncology, intraoperative imaging

## Abstract

**Background:** Fluorescence-guided surgery (FGS) using 5-aminolevulic acid (5-ALA) is a widely used strategy for delineating tumor tissue from surrounding brain intraoperatively during high-grade glioma (HGG) resection. 5-ALA reaches peak plasma levels ~4 h after oral administration and is currently approved by the FDA for use 2–4 h prior to induction to anesthesia.

**Objective:** To demonstrate that there is adequate intraoperative fluorescence in cases undergoing surgery more than 4 h after 5-ALA administration and compare survival and radiological recurrence to previous data.

**Methods:** Retrospective analysis of HGG patients undergoing FGS more than 4 h after 5-ALA administration was performed at two institutions. Clinical, operative, and radiographic pre- and post-operative characteristics are presented.

**Results:** Sixteen patients were identified, 6 of them female (37.5%), with mean (SD) age of 59.3 ± 11.5 years. Preoperative mean modified Rankin score (mRS) was 2 ± 1. All patients were dosed with 20 mg/kg 5-ALA the morning of surgery. Mean time to anesthesia induction was 425 ± 334 min. All cases had adequate intraoperative fluorescence. Eloquent cortex was involved in 12 cases (75%), and 13 cases (81.3%) had residual contrast enhancement on postoperative MRI. Mean progression-free survival was 5 ± 3 months. In the study period, 6 patients died (37.5%), mean mRS was 2.3 ± 1.3, Karnofsky score 71.9 ± 22.1, and NIHSS 3.9 ± 2.4.

**Conclusion:** Here we demonstrate that 5-ALA-guided HGG resection can be performed safely more than 4 h after administration, with clinical results largely similar to previous reports. Relaxation of timing restrictions could improve procedure workflow in busy neurosurgical centers, without additional risk to patients.

## Introduction

Maximal and safe resection has been established as the initial standard of care for the treatment of high-grade gliomas (HGG) (1–5). Complete resection of the contrast-enhancing tumor (CRET) has been associated with prolonged survival for patients with the most common HGG, glioblastoma (6, 7). Due to the propensity of HGGs involving eloquent regions of the brain, maximal safe resection poses intraoperative challenges for tumor surgeons (8). As a surgical adjunct, the use of fluorescence-guided surgery (FGS) provides surgeons with improved visualization of brain tumors and the infiltrative margin. The use of FGS has been well-studied in the use of HGG resection over the past 20 years and has shown to be an effective tool for resection of HGGs (9, 10).

Multiple fluorophore agents have been studied in the use of FGS for HGGs and each come with their own advantages and disadvantages (11). The most commonly studied fluorophores are 5-aminolevulinic acid (5-ALA), fluorescein and indocyanine green (ICG) (11). 5-ALA is the most widely studied agent for FGS of HGG, and is currently the only agent (Gleolan^®^) that is approved by the US Food and Drug Administration (FDA) for glioma surgery (9). Administered as an oral solution, 5-ALA is metabolized in the heme biosynthesis pathway to protoporphyrin (PpIX), which accumulates intracellularly in tumor cells (12), absorbing light between 375 and 440 nm and emitting violet-red fluorescence (640–710 nm) (13). 5-ALA has been previously shown in multicenter studies to be safe and effective, with minimal associated side effects (9, 14). Since the completion of the first phase III randomized controlled trial (RCT) for FGS showing improved progression-free survival (PFS) and greater overall tumor resection following FGS (9), 5-ALA has been used broadly across Europe and other countries throughout the world. However, 5-ALA (Gleolan^®^) only recently has been approved by the FDA in 2017 (15), and is currently being used by neurosurgeons throughout the country.

As part of the FDA approval of 5-ALA (Gleolan^®^ NX Development Corporation) for glioma surgery, the recommended usage in the label is stated as an “oral dose of ALA HCL solution of 20 mg/kg body weight, administered 3 h (range 2–4 h) prior to induction of anesthesia (16).” These recommendations were established after the RCT led by Stummer et al. (9). The timing of 5-ALA administration was based on rodent experiments in which a fluorescence peak was observed 6 h after administration (17). Oral administration at 3 h (2–4 h) was recommended in order to permit time for anesthesia, positioning, and craniotomy prior to peak intraoperative PpIX fluorescence (9, 10). Recently, however a study by Kaneko et al. found that maximal concentrations of fluorescence intensity were observed after 7–8 h following 5-ALA administration (18), calling for later administration than established in prior studies. In this study, we aim to study a population of patients undergoing glioma surgery beyond the 4 h window (>6 h) following 5-ALA administration as described in the FDA label to determine intraoperative fluorescence, as well as clinical and radiographic outcomes of patients undergoing FGS.

## Methods

### Patient Inclusion

Institutional Review Board approval was obtained with waiver of patient informed consent due to the retrospective nature of the study. For the purposes of this study, all patients receiving 5-ALA for resection of radiographic high-grade glioma (HGG) were screened at two separate institutions between 2017 and 2020. Patients were included if they received anesthesia induction more than 4 h following 5-ALA administration. Patients were excluded if they received anesthesia induction within 4 h of 5-ALA, or if they had non-HGG tissue upon histopathology. Patient demographic variables including age, sex, and preoperative functional status as measured by modified Rankin scale (mRS) were collected. Treatment variables included chemotherapy or radiotherapy. Operative variables including anesthesia induction time, incision time, procedure finish time, and time to extubation were collected. Outcome variables included postoperative neurological deficits, mRS scores, Karnofsky Performance Scores (KPS), National Institutes of Health Stroke Scale (NIHSS), 6-month progression-free survival (PFS), and time until death.

### Volumetric Analysis

Volumetric analysis from preoperative and postoperative MR imaging was prospectively collected for all patients. Volumetric measurements were made using Olea Sphere (v. 2.3, Olea Medical Solutions, La Ciotat, France) at one institution and Brainlab Elements (Brainlab, Munich, Germany) at the other. Regions of contrast-enhancement were measured in preoperative scans, immediate postoperative scans, and MRIs 6 months following surgery independently by three of the authors (R.B., P.S., S.H.), blinded to the clinical characteristics of the patient cohort, one being a neuroradiology fellow at the time of assessment. One patient volume required confirmation by a senior author (A.R.) who was not previously blinded to patient characteristics.

### Statistical Analysis

Clinical, operative, and radiographic pre- and post-operative characteristics are presented as frequencies and percentages for categorical variables, and means and standard deviations for continuous variables. Descriptive statistics were used to analyze both categorical and continuous variables. All statistical analyses were performed on Statistical Analysis Software version 9.4 (SASv9.4 - Cary, NC). Significance for all statistical testing was determined by *p* < 0.05.

## Results

### Patient Demographics and Tumor Characteristics

A total of 16 patients met the inclusion criteria (Table 1), 6 of them female (37.5%), with a mean (SD) age of 59.3 ± 11.5. Preoperative mean modified Rankin score (mRS) was 2 ± 1. All 16 patients received chemotherapy and/or radiation therapy in addition to resection for treatment of their brain tumor. Average tumor volume was 24.9 ± 24.6 cc. Fifteen (93.8%) patients' tumors were diagnosed as glioblastoma (GBM) on histopathology (one patient had anaplastic astrocytoma), 13 (86.7%) were IDH1 wild-type and 2 (13.3%) were IDH1 mutants. Nine (56.3%) tumors were primary and seven (43.7%) were recurrent HGGs (Table 2). Twelve (75%) tumors involved eloquent cortex. The mean extent of resection (EOR) was 91.5%, with 13 (81.3%) cases having residual contrast enhancement on postoperative MRI, and a mean residual volume of 1.16 ± 1.11 cc.

**Table 1:** Patient baseline characteristics.

**Variable**	**Patients, % (*N* = 16)**
Age, years (mean, SD)	59.3 ± 11.5
Female sex	6 (37.5)
Preoperative mRS (mean, SD)	2 ± 1
5-ALA dose	
20 mg/kg	16 (100)
Timing from 5-ALA, hours:min (mean, SD)	
Time to intubation	7:04 ± 5:34
Time to incision	8:26 ± 5:43
Time to procedure finish	12:46 ± 6:35
Time to extubation	13:06 ± 6:34
Intraoperative Fluorescence	
Signal presence	16 (100)

**Table 2:** Lesion characteristics.

**Variable**	**Patients, % (*N* = 16)**
Lesion pathology	
Glioblastoma multiforme	15 (93.7)
Anaplastic astrocytoma	1 (6.3)
Ge IDH mutation	2 (12.5)
Tumor location^*^	
Frontal	3 (18.8)
Temporal	8 (50)
Parietal	4 (25)
Occipital	4 (25)
Eloquent cortex	12 (75)
Lesion depth from cortex (mm)	6.8 ± 7.2
Lesion maximal diameter, mm (mean, SD)	49.4 ± 17.2
Lesion volume (mL) [volumetric]	24.9 ± 24.6

* *Three lesions occupied both parietal and occipital lobes*.

### 5-ALA Administration and Tumor Fluorescence

**Figure 1:**
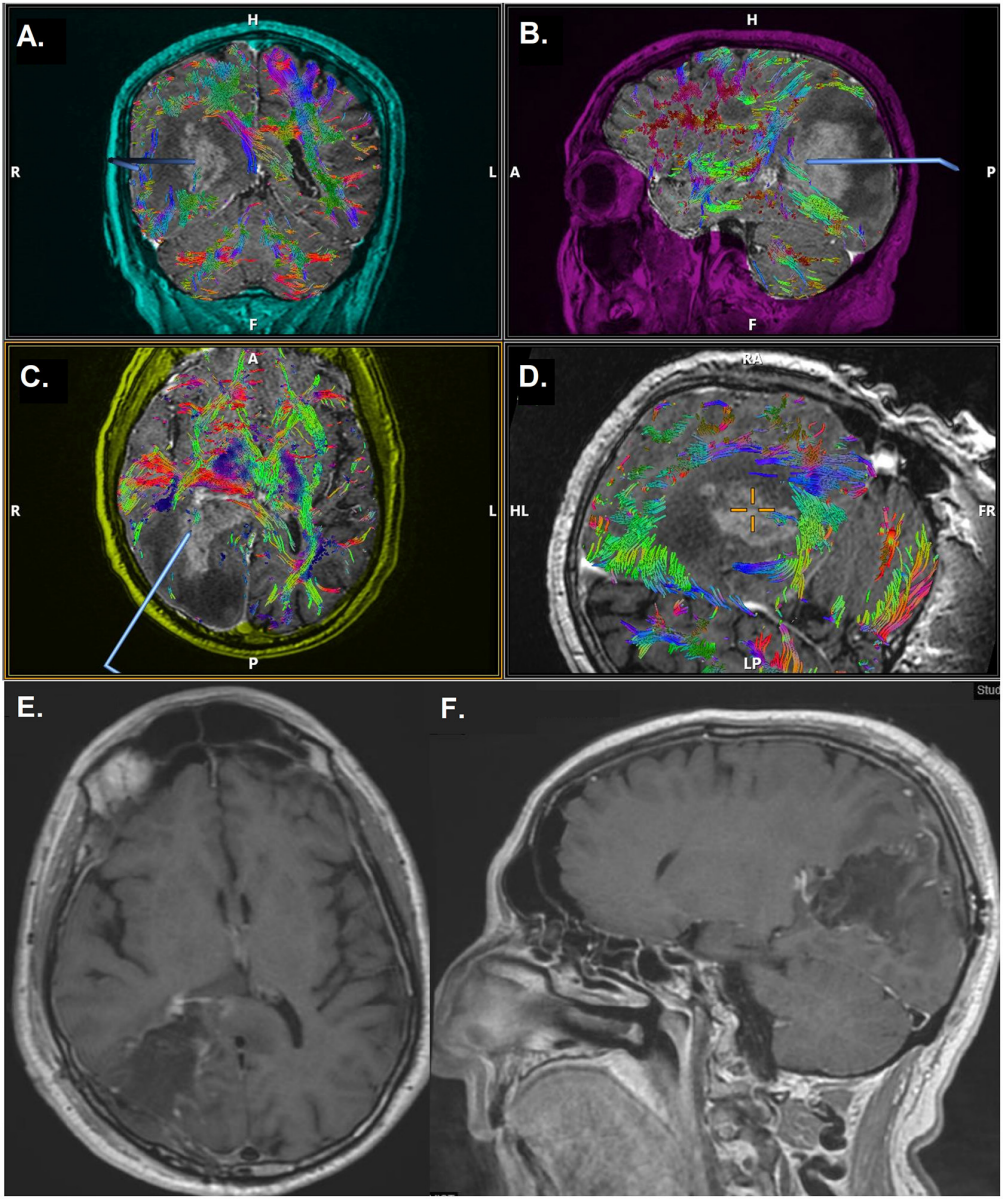
Case demonstration of a 69-year-old male with glioblastoma multiforme, undergoing 5-ALA FGS. Time from 5-ALA administration to anesthesia induction was 7 h and 48 min, time to incision was 9 h and 4 min and time to closure was 12 h and 14 min. **(A–D)** Preoperative MRI with DTI. Axial **(E)** and sagittal **(F)** postoperative MRI scan, after 5-ALA FGS, demonstrating gross total resection of the lesion. 5-ALA, 5-aminolevulinic acid; FGS, fluorescence-assisted glioma surgery; MRI, magnetic resonance imaging; DTI, diffusion tensor imaging.

**Figure 2:**
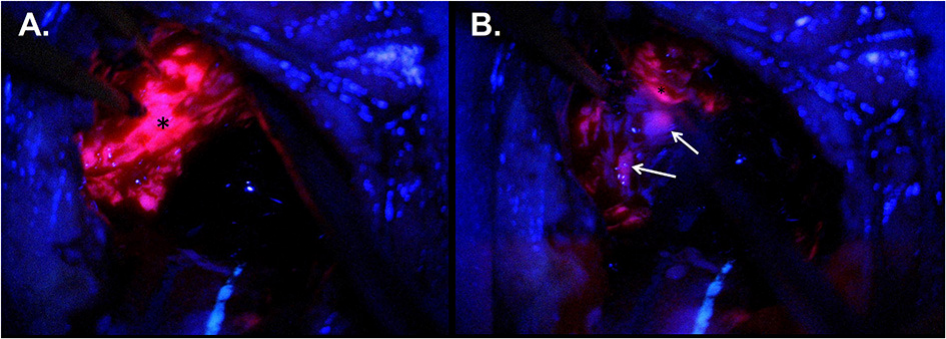
Intraoperative imaging for case demonstration patient. **(A)** Tumor bulk fluorescence after 5-ALA administration (asterisk). **(B)** Infiltrative margin fluorescence after 5-ALA administration (white arrows).

All patients were dosed with 20 mg/kg 5-ALA the morning of surgery. The mean time from 5-ALA administration to incision was 507 ± 344 min and time to closure was 767 ± 395, therefore the fluorescence guided surgery was performed after >6 h in all cases. The longest time between 5-ALA administration and anesthesia induction was 27 h and 46 min. This patient received 5-ALA prior to scheduled surgery but was found to have a fever, had a full workup over the ensuing day, then proceeded to have FDG surgery the next day, with adequate fluorescence intraoperatively. All cases had adequate intraoperative fluorescence. No patients in the series experienced 5-ALA-related toxicity. All sixteen patients' tumors demonstrated intraoperative fluorescence *in situ* (Figures 1, 2).

### Patient Outcomes

**Figure 3:**
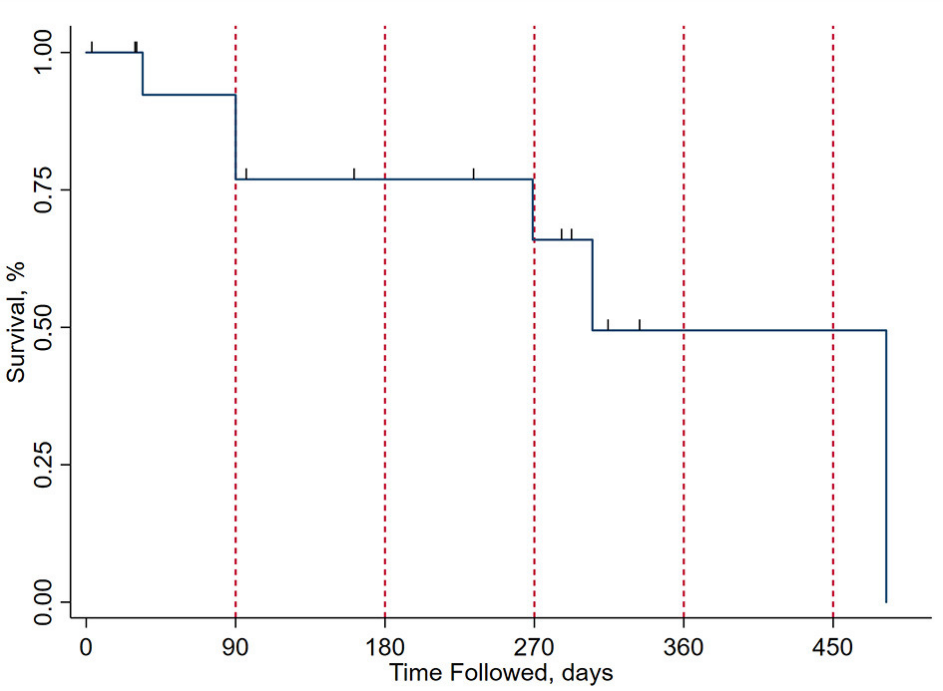
Kaplan-Meier curve for survival after surgical excision on the present cohort. The x axis represents time from surgical excision in days, with red vertical dashed lines at the 3-, 6-, 9-, 12-, and 15-month marks. The y axis represents the percentage of patients surviving at each time point. Drop points in the curve represent patient mortality and the short vertical lines represent subject concealment from further calculations due to end of follow-up, with patients either lost to follow-up or being operated on recently from the time of analysis.

Five (26.7%) patients had postoperative neurological deficits, defined as decrements in the NIHSS more than one point during the initial hospitalization (Table 3). Mean progression-free survival was 5 ± 3 months. During the study period, 6 (37.5%) patients died, mean mRS is 2.3 ± 1.3, mean Karnofsky score was 71.9 ± 22.1, and mean NIHSS was 3.9 ± 2.4. Six-month progression-free survival was seen in 46.2% (6) patients. Overall survival following surgery was 6.3 ± 4.9 months (Figure 3).

**Table 3:** Radiographic and clinical patient outcomes.

**Variable**	**Patients, % (*N* = 16)**
5-ALA-related toxicity	0 (0)
Residual contrast enhancement on postoperative MRI	13 (81.3)
Residual volume, cc (mean, SD)	1.16 ± 1.11
Percent extent of resection (EOR, mean, SD)	91.5 ± 10.7
Chemotherapy or radiation received	15 (93.8)
Total clinical follow-up, months (mean, SD)	5.7 ± 4.4
New neurological deficit	4 (26.7)
mRS	2.3 ± 1.3
Karnofsky score	71.9 ± 22.1
NIHSS	3.9 ± 2.4
Total radiographic follow-up, months (mean, SD)	3.8 ± 3.1
Radiographic recurrence or progression	8 (50.0)
Time to recurrence or progression, months (mean, SD)^$^	5 ± 3
Mortality	6 (37.5)
Time to mortality, months (mean, SD)^#^	6.3 ± 4.9
6-month progression-free survival^@^	6 (46.2)

*Missing data*:

$ *Out of 4 patients that had recurrence/progression*.

# *Out of 3 patients that died*.

@ *Missing 2 patients operated on <6 months ago*.

## Discussion

5-ALA is a well-studied intraoperative adjunct for the resection of HGGs that helps differentiate tumor from surrounding brain parenchyma and has been shown to improve gross total resection rates as well as overall patient survival (9, 19–32). Despite its widespread use, limited data exists regarding the best timing for administration of 5-ALA prior to surgery. The current guidelines set forth by the Food and Drug Administration (FDA) state that the substance should be administered “3 h (range 2–4 h) before anesthesia (16).” However, the exact fluorescence kinetics regarding PpIX accumulation within tumor tissue are currently unclear. In the current study, the safety and efficacy of 5-ALA use beyond the established time of administration was assessed. In patients administered 5-ALA over 6 h prior to the start of tumor resection, 5-ALA was not only safe to use during this time window, but also efficacious in both in diagnostic accuracy and the extent of resection.

The currently limited data regarding fluorescence kinetics of 5-ALA seem to indicate a more delayed fluorescence peak within HGGs than previously thought. In an early study utilizing an orthotopic brain tumor model, the maximum fluorescence intensity was shown to be around 6 h after administration (17). The recommended time of administration used in the summary of the product characteristics (SPC) for FDA approval (Gleolan^©^) was based upon animal studies showing that fluorescence peaked 6 h after administration (33). Extrapolated from these experiments, the parameter of administration of 3–4 h prior to anesthesia would allow time for operating room set up and resection in ample time for peak fluorescence during tumor resection (18). As a result, 5-ALA clinical trials, including the RCT, have utilized this parameter (9, 19–32). In the FDA New Drug Application (NDA), six clinical trials were included that used this parameter, all of which were conducted in Germany (34). Prior to this study, only one clinical trial recommended administration of 5-ALA 3–5 h prior to surgery. No studies have assessed a longer time interval.

Other studies have suggested that 5-ALA induced fluorescence may peak beyond the previously studied time window of 2–4 h. Studies measuring PpIX concentration in other parts of the human body have shown a peak in the plasma to be around 8 h (32), and in the skin to be 6.5 to 9.8 h (35). In a later study of 201 samples from 68 patients, Kaneko et al. investigated the time dependency of protoporphyrin IX (PpIX) by measuring fluorescence intensity in tumor biopsy samples at various time points during HGG surgery (18). The authors recorded the time-fluorescence curves in their ex-situ study, demonstrating that the peak intensity may be 7–8 h after 5-ALA administration, and suggested that 5-ALA be administered earlier than what is currently suggested by the FDA, specifically 4–5 h prior to anesthesia induction (18). In line with their work, we found that there was adequate intraoperative fluorescence to guide HGG resection in all our cases that were induced to anesthesia more than 4 h after imbibing 5-ALA. Our results confirm that tumor fluorescence is even present more than 24 h after oral administration in some cases.

The identification of the ideal timing of 5-ALA administration prior to surgery is of high importance for multiple reasons. In everyday practice, where operations may be postponed for various logistical reasons (staffing, cleaning of the operating room, emergent cases), it would be useful to know if any such delay could adversely affect intraoperative fluorescence and, possibly, patient outcomes. Individual institutions and surgeons should account for the time needed for exposure or mapping, and plan accordingly. For tumors known to demonstrate less fluorescence (e.g., WHO grade III gliomas), for tumor margins and in deep-seated gliomas, resection during the peak of fluorescence intensity would be ideal. Another important aspect regarding administration of 5-ALA is the timing of 5-ALA and PpIX clearance from the tumor bed. Interestingly, glioblastomas seem to maintain up to 65% of their maximal fluorescence even 10 h after 5-ALA administration (18), confirming that fluorescence clearance may be much slower than its accumulation. Kaneko et al. found that tumor type may predict clearance time of fluorescence (18), which may advocate for further investigation on establishing different time periods for administration based upon pathology. Additionally, the infiltrative tumor margin may have a later peak at around 8–9 h, further corroborating the need for more delayed administration (18, 33). However, the exact timing of fluorescence clearance from the tumor bed is not currently known and further study is warranted to determine the true extent of the 5-ALA time window in order to demonstrate *in situ* results regarding when fluorescence is no longer visible.

The current study did not demonstrate any concerns with patient safety, and had comparable outcomes to prior studies. Of the 16 patients included between two centers, there were no photosensitivity reactions or other 5-ALA-related toxicities noted. The average extent of resection was over 90%, defined as a gross total resection. This compares to other observational and clinical trials showing GTR rates ranging between 25 and 94% with 5-ALA (9–11). In terms of patient outcomes, patients saw an average progression-free survival (PFS) rate of 5 months, with a 6-month PFS rate of 46.2%, which is comparable to previous studies showing a 6-month PFS rate of 46% (22) and average PFS of 8.6 months (36). In our cohort, overall survival was 6.3 ± 4.9 months, which is less than prior studies on 5-ALA FGS (9–11), and may be explained by the patients in the current study. Twelve (75%) patients had tumors in eloquent cortex, which may be less amenable to maximal resection, therefore conferring a lower overall survival. Additionally, multiple patients in the cohort had surgery for recurrent GBM, which is also associated with a lower overall survival rate (37, 38). While this cohort is smaller than prior clinical trials, it did not appear that earlier administration of 5-ALA had an impact on both surgical factors and patient outcomes.

## Limitations

Despite the strengths of this study, there are limitations that must be addressed. The most significant limitation to the present study is the design. As a small, retrospective, single-arm study, the level of evidence is limited, and patient and clinical variables were not able to be controlled. Historical controls were used rather than a randomized control group, and therefore findings from our study may only be assessed as associations, rather than causational. Additionally, the current study was performed at two centers over a 2-year period, and therefore the results may not be representative of other centers. Intraoperative fluorescence was assessed by the primary surgeon and verified by a second surgeon, and no quantified data was obtained, therefore, there may be an observer bias as there were no controls and subjects were not blinded. To address many of these limitations, a larger, multicenter randomized controlled trial is warranted to determine the safety and efficacy of 5-ALA administration at a longer time window between administration and surgical resection.

## Conclusion

In this preliminary case series, we demonstrate that 5-ALA FGS can be carried out safely more than 6 h after administration of the substance, with clinical results being largely similar to previous reports. The relaxation on restrictions regarding timing of surgery after 5-ALA administration could have positive implications on procedure scheduling and workflow in busy neurosurgical centers, without any additional risk to patient safety and surgical outcomes.

## Data Availability Statement

The raw data supporting the conclusions of this article will be made available by the authors, without undue reservation.

## Ethics Statement

The studies involving human participants were reviewed and approved by Mount Sinai Institutional Review Board. Written informed consent for participation was not required for this study in accordance with the national legislation and the institutional requirements.

## Author Contributions

All authors listed have made a substantial, direct and intellectual contribution to the work, and approved it for publication.

## Conflict of Interest

CH is a consultant for NX Development Corporation (NXDC) and Synaptive Medical. NXDC, a privately held company, markets Gleolan (5-ALA, aminolevulinic acid hydrochloride). Gleolan is an optical imaging agent approved for the visualization of malignant tissue during glioma surgery. CH is a consultant for NXDC and receives royalty payments for the sale of Gleolan. CH receives financial compensation as a consultant and lecturer for Synaptive (manufacturer of the 3D Synaptive MODUS V device). He has also received speaker fees by Carl Zeiss and Leica. The remaining authors declare that the research was conducted in the absence of any commercial or financial relationships that could be construed as a potential conflict of interest.
